# T_2_* weighted Deconvolution of NMR Spectra: Application to 2D Homonuclear MAS Solid-State NMR of Membrane Proteins

**DOI:** 10.1038/s41598-019-44461-3

**Published:** 2019-06-03

**Authors:** Manu V. S., Tata Gopinath, Songlin Wang, Gianluigi Veglia

**Affiliations:** 1 Department of Biochemistry, Molecular Biology and Biophysics, University of Minnesota, Minneapolis, MN USA; 2 Department of Chemistry, University of Minnesota, Minneapolis, MN USA

**Keywords:** Biophysics, Structural biology

## Abstract

2D homonuclear NMR spectroscopy is an essential technique to characterize small and large molecules, such as organic compounds, metabolites, and biomacromolecules at atomic resolution. However, for complex samples 2D homonuclear spectra display poor resolution, making spectral assignment very cumbersome. Here, we propose a new method that exploits the differential T_2_* relaxation times of individual resonances and resolves the 2D NMR peaks into pseudo-3D spectra, where time is the 3^rd^ dimension. T_2_* weIghted DEconvolution or TIDE analyzes individual free induction decays (FIDs) and dissects them into sub-FIDs that are transformed into pseudo-3D spectra combining Fourier transformation and covariance NMR. TIDE achieves higher resolution and sensitivity for NMR spectra than classical covariance NMR reducing offset-dependent artifacts. We demonstrate the performance of TIDE for magic angle spinning (MAS) [^13^C,^13^C]-DARR NMR spectra of single- and multi-span membrane proteins embedded in lipid bilayers. Since TIDE is applicable to all type of homonuclear correlation experiments for liquid and solid samples, we anticipate that it will be a general method for processing NMR data of biomacromolecules, complex mixtures of metabolites as well as material samples.

## Introduction

In classical multidimensional NMR spectroscopy, the free induction decay (FID) is recorded as complex time-domain signals and converted into frequency-domain spectra using Fourier transform (FT)^1–3^. First introduced by Jeener^2^ and Ernst^3^, 2D NMR spectroscopy remains one of the most widely used techniques to analyze small and large molecules at atomic resolution both for solution and solid-state NMR (ssNMR). However, the complexity of NMR spectra often calls for advanced processing techniques such as linear prediction and maximum entropy reconstruction to enhance the spectral quality, *i*.*e*., resolution and sensitivity^4–6^. In 2004, Bruschweiler and Zhang introduced covariance NMR^7,8^ as a way to obtain higher quality of homonuclear correlation spectra of small molecules and metabolites. In these past years, covariance NMR has also been applied to homonuclear 2D, 3D, and 4D solution^9,10^ and ssNMR spectra of large proteins^11,12^. Takegoshi and co-workers implemented covariance NMR for hetero-nuclear correlations experiments^13^, widening the number of applications for this powerful technique. Since it entails only the real part of the FID in the indirect dimension, covariance NMR reduces the total experimental time over classical FT by 50%. Although covariance NMR increases sensitivity of 2D homo- and hetero-correlation spectra, it falls short to improve the resolution of crowded spectra.

Here we present a new method that further enhances simultaneously both spectral resolution and sensitivity of 2D NMR data sets. T_2_* weIghted Deconvolution (TIDE) separates NMR peaks depending on their intrinsic transverse relaxation times (T_2_*). TIDE-processed spectra consist of two chemical shift dimensions resolved as a function of the time increments according to their T_2_* in a pseudo-3D mode. We demonstrate the performance of TIDE for magic angle spinning (MAS) [^13^C,^13^C]-DARR^14^ spectra of single- and multiple-pass membrane proteins. Using TIDE, we achieved partial or complete separation of overlapped resonances in the spectra of these membrane proteins, improving dramatically both resolution and sensitivity with respect to the classical FT and covariance NMR.

## Results

### Theory of the TIDE method

In a standard 2D FT-NMR experiment, the time domain signals, S (*t*_1_, *t*_2_), are transformed into frequency-domain, S (*ω*_1_, *ω*_2_), using FT for both dimensions. For covariance NMR (Fig. S1**)**, the time-domain signals, S (*t*_1_, *t*_2_), are first Fourier transformed along the direct dimension *t*_2_, obtaining S (*t*_1_, *ω*_2_). Subsequently, the covariance or cross-correlation spectrum is calculated from the *t*_1_-encoded 1D spectra S (*t*_1_, *ω*_2_) using:

1 $${{\rm{C}}}_{{\rm{ij}}}=\frac{1}{{{\rm{N}}}_{1}-1}\,\sum _{{\rm{k}}=1\,}^{{N}_{1}}({\rm{S}}(k,\,{\rm{i}})-\langle {\rm{S}}({\rm{i}})\rangle )\,({\rm{S}}(k,\,{\rm{j}})-\langle {\rm{S}}({\rm{j}})\rangle )$$

where i and j represent row and column index of the covariance matrix, respectively, *k* is the index of the FIDs in the *t*_1_ dimension, N_1_ is the total number of time points in the indirect dimension and $\langle {\rm{S}}({\rm{i}})\rangle =1/{{\rm{N}}}_{1}\sum _{k\,}{\rm{S}}(k,{\rm{i}})$ is the mean of *i*^th^ column vector of S. The cross correlation spectrum (R_ij_) is evaluated using ${R}_{{\rm{ij}}}={C}_{ij}/\sqrt{{C}_{ii}.{C}_{jj}}$.

**Figure 1:**
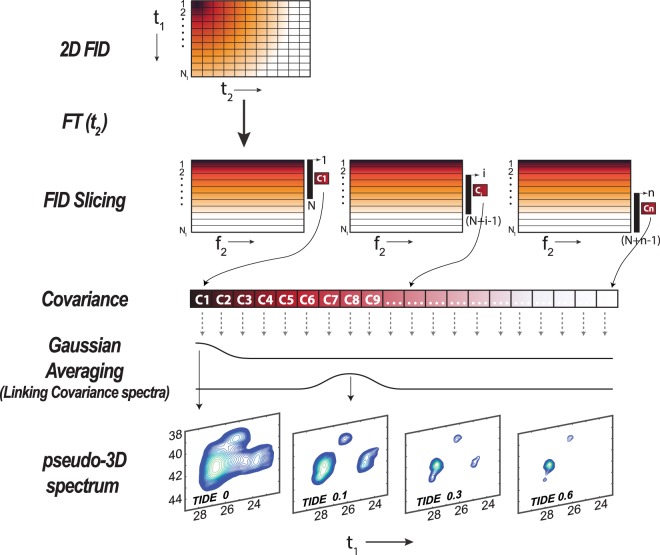
Schematic of TIDE method. The complex 2D FID matrix is FT along direct (*t*_2_) dimension. The FIDs in the indirect dimension (t_1_) are sliced prior to covariance calculations. The array of covariance spectra from the sub-FIDs are multiplied for a Gaussian function prior to being visualized into a pseudo-3D spectrum, where the pseudo-third dimension is time.

For TIDE (Fig. 1), after the first FT in the *t*_2_ dimension, the *t*_1_ FIDs, S [*t*_1_(1 to N_1_), *ω*_2_], are dissected into a series of *n* sub-FIDs (Fig. S2):

2 $$S[{t}_{1}(1\,to\,{N}_{1}),{\omega }_{2}]=\{{S}_{1}[{t}_{1}(1\,to\,N),{\omega }_{2}],{S}_{2}[{t}_{1}(2\,to\,N+1),{\omega }_{2}]\ldots {S}_{n}[{t}_{1}(n\,to\,N+n-1),{\omega }_{2}]\}$$

where N is number of *t*_1_ points in each sliced FID and N + n − 1 = N_1_, which indicates the total number of *t*_1_ points. The dissection of the initial FID into sub-FIDs separates the signal of short-lived resonances. In fact, as sub-FID index(*i*) increases, the relative amplitude for the long-lived signals increases, while the short-lived coherences die off. Subsequently, the S_i_ series of sub-FIDs are transformed into frequency domain using covariance NMR. Note that if we used FT in this step, which relies on initial phases of the signals in the *t*_1_ dimension, we would introduce first-order phase distortions in the spectra resulting from the missing time points and dwell time in the sub-FIDs. In contrast, we opted for covariance transformation, which avoids phase distortions. The 2D covariance spectrum C_i_ (ω_1_, ω_2_) is calculated for each sub-FID using Eq. 1. The pseudo-3D TIDE spectrum is generated by performing a Gaussian averaging over all the C_i_ (ω_1_, ω_2_) spectra:

3 $${TID}{{E}}_{p}=\sum _{i=1}^{n}{C}_{i}({\omega }_{1},\,{\omega }_{2})\times {G}_{i}(p)$$

where *p* indicates the plane number in the TIDE pseudo 3^rd^ dimension and *G* is the Gaussian distribution defined over the covariance spectra indices, and σ^2^ is the variance:

4 $${G}_{i}(p)=\frac{1}{{\rm{\sigma }}\sqrt{2{\rm{\pi }}}}{{\rm{e}}}^{-{({\rm{i}}-{\rm{p}})}^{2}/2{{\rm{\sigma }}}^{2}}$$

Note that, even though we are dividing each FID in the t_1_ dimension to create a series of sub-FIDs, the final TIDE spectrum contains all the points for each FIDs that are linked via Gaussian averaging.

The Gaussian averaging links all the Covariance spectra generated from the sub-FIDs. In addition, the Gaussian averaging reduces the frequency dependent artifacts in the spectra and hence improves the quantitative nature of the peaks. The variance dictates the width of the Gaussian function and is typically set to 10% of the total number of the sub-FID Covariance (Ci). σ^2^ is treated as a variable by the algorithm and will vary based on the size of indirect time points. In order to demonstrate the impact of Gaussian averaging, we evaluated the phase and frequency responses of covariance of sine waves. Varying the frequency of the sine wave imparts a covariance-frequency oscillation (Fig. S3A). The amplitude of the oscillation is highest near zero frequency and the oscillating pattern repeats itself every integer multiple of the Nyquist frequency. Changing the phase of the sine wave creates a periodic covariance-phase oscillation as shown in Fig. S3B. The Gaussian averaging over this phase oscillation reduces the covariance-frequency oscillation improving the frequency response. A detailed stepwise procedure for TIDE processing is explained in Supporting Information.

### TIDE increases both resolution and sensitivity of 2D homonuclear correlation spectra

To demonstrate the advantages of TIDE in terms of sensitivity, we tested its performance on a series of [^13^C,^13^C]-DARR experiments carried out on a U-^13^C, ^15^N labeled N-acetyl-Val-Leu (NAVL) dipeptide recorded with a mixing time of 10 ms and different signal-to-noise (S/N) ratios, which were obtained varying the angle (θ) of the initial excitation pulse on the ^1^H channel (Fig. S7). The highest S/N ratio was obtained for θ = 90° and the peak intensities were normalized to 100%. The spectra obtained with θ = 5°, 2°, and 1° gave relative sensitivities of 8.5, 3.5, and 1.8%, respectively (Fig. S9A). The 2D spectra were processed using TIDE and FT. A comparison of the processed spectra is reported in Fig. S9B and C. As expected, the cross-peaks of the FT spectra follow closely the S/N ratio and become almost undetectable for the experiment acquired with θ = 1°. In contrast, the TIDE spectrum shows cross correlations even at low S/N ratio (~5). For S/N ratios lower than 5, TIDE introduces artifacts as shown in the Cα region of the spectrum. The latter becomes a significant problem when the experiments are carried out with small number of time increments in the indirect dimension.

We first tested the performance of TIDE over the original covariance NMR. To this extent, we analyzed the frequency response of the peak intensities using two simulated FIDs of 256 points, consisting of a single frequency resonance with the same signal and noise amplitudes, but different noise profiles (Fig. S4A). The first FID, FID-1, represents the decay of a single resonance, *R*_*a*_, and the second, FID-2, with different noise profile is the decay of *R*_*a*_′. We calculated the correlation coefficients between the two FIDs for a range of frequencies and generated a response plot (Fig. S4B). This response plot shows that TIDE does not introduce any frequency-dependent artifacts in the transformed spectra. We then repeated the same simulations for *R*_a_ and *R*_a_′ with a third frequency, *R*_b_, summed to FID-1 (Fig. S5). The covariance calculation between FID-1 and FID-2 gives cross-peaks at frequencies ω_2_ and ω_2_′ for the direct dimension. If *R*_*b*_ is present in FID-1, the correlation coefficient between the two FIDs is reduced (Fig. S5C). The decrease of the correlation coefficient between the two FIDs is modulated by the lifetime of *R*_*b*_ (T_2b_^*^). For T_2b_^*^ ≪ T_2a_^*^, *i*.*e*., *R*_*b*_ decays rapidly, the correlation coefficient increases. For instance, if T_2a_^*^ = 5 ms and T_2b_^*^ = 1 ms, the correlation coefficient increases up to 7% (Fig. S4C). A similar scenario can be envisioned for T_2b_^*^ ≫ T_2a_^*^. The resolution gain achievable with TIDE is even more apparent with a 2D homonuclear correlated data set. As an example, we simulated a short-lived resonance with T_2_ = τ overlapping with two correlated slowly relaxing resonances (T_2_ = 2τ) (Fig. S6). In the FT spectrum, it is possible to observe only one unresolved resonance. Analogously, in the first few planes of the pseudo-3D TIDE spectrum, only one unresolved peak is observed. However, by analyzing the following planes (*Movie* *1*. *avi*, Supporting Information) the low-intensity resonances appear (30^th^ plane) and are fully resolved and more intense in the 90^th^ plane (Fig. S6). Taken together, these simulations show that covariance NMR and TIDE give almost identical results for a spectrum with a single frequency, whereas TIDE outperforms covariance NMR when additional resonances are present in the FID.

### Application of TIDE to membrane proteins

The performance of TIDE can be appreciated for more heterogeneous systems such as membrane proteins embedded in lipid membranes. To illustrate this point, we analyzed the time-frequency distribution of an experimental FID using reduced interference distribution (RID, http://case.caltech.edu/tfr/). RID is a time-frequency analysis technique that defines the frequency content of time-dependent signals. Fig. S8A displays the RID output for the first FID of the [^13^C,^13^C]-DARR spectrum of the single-pass membrane protein phospholamban (PLN)^15^ embedded in 1,2-dimyristoyl-sn-glycero-3-phosphocholine (DMPC) lipid bilayers. The FID of PLN can be shown as a time-resolved pseudo-2D, where the acquisition time constitutes the first dimension and the 1D spectra the second dimension (Fig. S8A). This pseudo-2D or time-resolved 1D spectrum was obtained by removing iteratively the first time point of the FID and plotting the absolute values of the corresponding FT. The pseudo-2D illustrate the differential T_2_* of each individual ^13^C resonance.

Due to dilute sample conditions and primary sequence redundancies, the [^13^C,^13^C] DARR spectra of membrane proteins such as PLN suffer of poor sensitivity, and resolution. We applied TIDE to the [^13^C,^13^C] DARR spectra of two single-pass membrane proteins, phospholamban (PLN)^16^ and sarcolipin (SLN)^17^ as well as to SatP, a six-transmembrane acetate-succinate permease from *E*. *coli*^18–20^. The DARR spectra of PLN and SLN processed with FT are taken from our previous work^16,17,21^.

**Figure 2:**
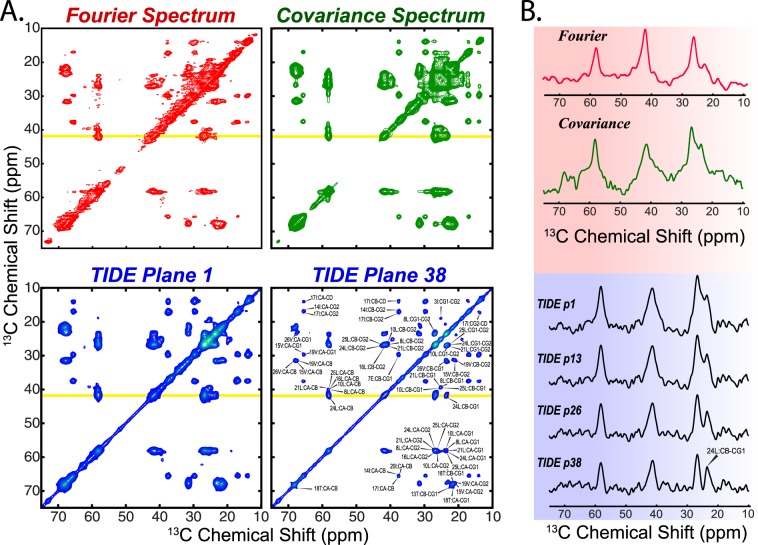
(**A**) Comparison of the [^13^C,^13^C]-DARR of sarcolipin (SLN) using FT (red), Covariance NMR (green) and TIDE (blue). (**B**) 1D slices extracted from the above spectra along the yellow line. The [^13^C,^13^C]-DARR experiment was acquired on a Agilent 600 MHz with 100 ms mixing time using a 3.2 mm bioMAS probe spinning at 12 kHz. The complete experimental parameters are listed in Table S1. The data sets were processed using Matlab (version 8.5) with an in-house written software. The original data published previously and reported here with permission (copyright Springer Nature) from Mote *et al*. [ref.^17^].

**Figure 3:**
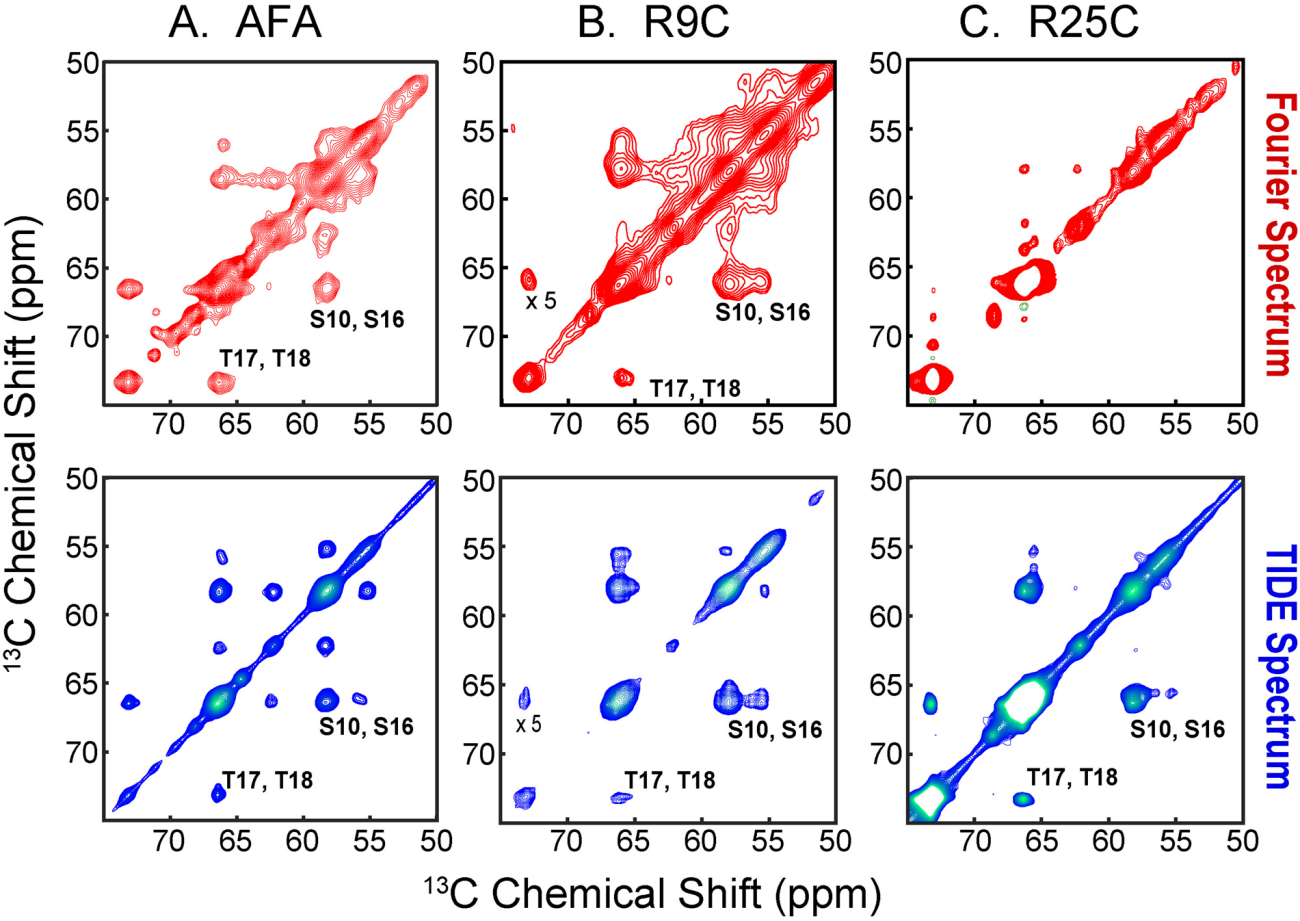
Comparison of the [^13^C,^13^C]-DARR of PLN^AFA^ (**A**), PLN^R9C^ (**B**) and PLN^R25C^ (**C**) processed using classical FT (red) and TIDE (blue). The [^13^C,^13^C]-DARR experiments on PLN and its mutant were acquired on a Agilent 700 MHz spectrometer. All DARR experiments were performed with 100 ms mixing time using a 3.2 mm bioMAS probe spinning at 12 kHz. The complete experimental parameters are listed in Table S1. The data sets were processed using Matlab (version 8.5) with an in-house written software. The original data published previously and reported here with permission (copyright Elsevier) from Nelson *et al*. [ref.^16^].

The comparison of U-^13^C,^15^N SLN [^13^C,^13^C] DARR spectra processed with FT, covariance NMR, and TIDE is shown in Fig. 2. Due to the severe overlap or missing peaks, the resonance assignment of SLN has been challenging in spite of its small size and required several selectively labeled samples^22^. With respect to FT, covariance NMR spectra display significantly higher S/N ratio and several peaks that were weak or missing in the classical processing are more intense. However, the magnetic equivalence of several resonances results in a significant spectral overlap. In addition, both FT and covariance NMR spectra display a marked broad base of the resonances, which is probably due to inhomogeneities and/or incomplete averaging of chemical shift anisotropy (CSA) and dipolar couplings (DC), which causes major resolution losses in the DARR spectra. In contrast, the TIDE planes (plane 1 and plane 38) reported in Fig. 2A show a significant enhancement of the S/N ratio and a concomitant higher resolution of resonances in the spectrum. CSA and DC broadening effects are ameliorated in the TIDE processing as shown in the *t*_1_-resolved planes, which enabled us to obtain well-separated peaks (Fig. 2B). Remarkably, the high resolution achieved in plane 38 of the pseudo-3D, made it possible to assign most of the SLN resonances. We also tested the performance of TIDE for DARR spectra of a 52 amino acid single-pass membrane protein, PLN, and its disease-linked mutants, PLN^R9C^ ^23^ and PLN^R25C^ ^24^ (Fig. S10). In lipid membranes, the cytoplasmic region of PLN (residues 1–25) undergoes a slow conformational exchange between an ordered T state (membrane bound), and a sparsely populated R state (membrane detached)^22^. The DARR spectra show both populations. Specifically, in the monomeric form of PLN (PLN^AFA^) the T and R state populations were estimated to be 97 and 3%, respectively. For the PLN^R9C^ mutant, the cytoplasmic region is predominantly in the membrane-bound state, whereas the PLN^R25C^ populates mostly the R-state^16^. The DARR experiment uses a cross polarization element to transfer the nuclear polarization and detects the more rigid, residues membrane-bound T state. In Fig. 3, we show portions of DARR spectra featuring Ser and Thr resonances that we use to estimate the extent of T state. The resonances of these residues are present for both PLN^AFA^ and PLN^R9C^, but are essentially undetectable in the FT spectra of PLN^R25C^ as the T state is sparsely populated. In contrast, TIDE processing enabled us to visualize even the small population of the T state for PLN^R25C^. A detailed discussion of PLN^AFA^, PLN^R9C^ and PLN^R25C^ can be found in our previous article by Nelson *et al*.^16^. The improved sensitivity and resolution are also apparent for DARR spectra used to detect inter-helical DC between the asymmetrically labeled protomers of the wild-type pentameric PLN^21,25^. The [^13^C,^13^C] DARR spectrum was recorded using 200 ms mixing time to detect long-range DC to define the Ile/Leu zipper holding together the pentameric assembly (Fig. S11). The experiment was acquired with only 50 points in the indirect dimension, which are at the limit of TIDE applicability. Nevertheless, the first plane of TIDE outperforms the FT spectrum as shown in both the 2D and 1D cross sections and we were able to resolve previously overlapped inter-protomer correlations. Finally, we compared the different processing methods on the [^13^C,^13^C]-DARR experiments carried out on the six-transmembrane domain SatP (Fig. S12)^18^. The TIDE processed spectra are significantly more intense and resolved than the corresponding FT spectra, in which several correlation peaks are obscured by fast relaxing resonances.

## Discussion

Since the incipit of NMR spectroscopy^26–28^, scientists have continued their quest for improving spectral resolution. A traditional way to improve spectral resolution is to add new dimensions, separating the overlapped signals based on their magnetic properties such as chemical shifts, J couplings, dipolar couplings *etc*. With our new method, we utilized a distinct feature of the FIDs, i.e., the differential T_2_* of the individual spins. This leads to a non-uniform distribution of nuclear resonance frequencies that is apparent for heterogeneous systems such as mixtures of metabolites, materials or large biomacromolecules. Campbell *et al*.^29^ exploited this phenomenon to filter out unwanted signals from 1D NMR spectra. Similar strategies were used for ligand binding studies^30^, small molecules spin-lattice relaxation measurements^31^, diffusion-edited drug binding^32^ and eliminate the signals of large macromolecules from spectra of metabolites^33^. Ding *et al*.^34^ performed T_2_ based filtering of multiple-quantum spectra in solids with strong dipolar interactions. This was achieved by adding a delay before the acquisition time. This approach enhances the resolution of ^1^H spectra, but decreases the sensitivity and causes phase and baseline distortions in the second dimension. In contrast to these methods, TIDE utilizes differential T_2_* of the spin systems to spectrally separate them and assign individual resonances. TIDE dissects each FID into sub-FIDs separating fast from slow relaxing spin systems and then reconstructs the entire spectrum into a pseudo-3D combining both FT and covariance NMR. In this way, TIDE extends the dimensionality of the 2D homonuclear correlation spectroscopy by retaining time-frequency information encoded in the FID. The pseudo-third dimension enables one to resolve overlapping peaks according to the transverse relaxation properties. Remarkably, TIDE reduces the offset dependent artifacts significantly, and hence, the first plane of TIDE is superior to the original covariance spectra. As for the recent modification of covariance NMR^35^, the peak intensities obtained with TIDE are not modulated by the carrier frequency. Therefore, TIDE-processed DARR cross-peaks can be binned and converted into ranges of distance constraints for structure calculations. Importantly, TIDE also preserves the original advantages of covariance NMR^7^. In fact, it can be applied to data sets acquired with standard pulse sequences without modifications of experimental parameters and requires only real points in the indirect dimension, reducing the experimental time by 50%. Another benefit shared by covariance NMR and TIDE is the improved resolution even in the presence of asymmetry in the peak intensities with respect to the diagonal. It has been shown that multidimensional solid-state NMR correlation spectra are inherently asymmetric, due to cross-polarization spin dynamics and complex motions^36^. Nonetheless, covariance processing improved dramatically the quality of the spectra of microcrystalline protein preparations^12^ and large membrane proteins^37^. A similar result was obtained by Lin and Opella, who used covariance processing for solid-state NMR spectra of oriented membrane protein samples^11^. In this latter case, the lower intensity of the signals and the asymmetry of the peaks due to spin diffusion further jeopardize the quality of the spectra. Nonetheless, covariance processing offered a higher quality spectra. As for covariance NMR, TIDE processed spectra show an increase in both resolution and signal-to-noise ratio. The most remarkable application is the observation of minor population in the DARR spectra of PLN mutants, which were completely missed in our earlier 2D FT processing^16^. Nonetheless, the most significant advancement accomplished by TIDE is the introduction of the pseudo-third dimension, which further separates the resonances of highly overlapped, heterogeneous systems such as membrane proteins. We believe that TIDE will make it possible to simplify the NMR spectra of these systems as well as other heterogenous samples such as polymers, complex mixtures of metabolites as well as other materials.

## Material and Methods

### Sample preparation

PLN, SLN, and SatP were expressed recombinantly in *E*. *coli* bacteria. For PLN and SLN, we used a maltose binding protein as a fusion. The complete expression and purification protocols were reported previously^38^. For the succinate-acetate permease (SatP), we used a SUMO fusion protein to enable the purification. The complete protocol for SatP purification was reported by Gopinath *et al*.^39^. The NAVL sample was synthesized and crystallized according to the preparations reported by Tenkortenaar *et al*.^40^.

### NMR spectroscopy

NMR experiments were performed on 600 and 700 MHz Agilent solid-state spectrometers as well as 700 MHz Bruker solid-state spectrometer. For the MAS experiments about 1.5 mg of SLN, 2 mg of PLN and 8 mg SatP were reconstituted into 1,2-dimyristoyl-sn-glycero-3-phosphocholine (DMPC, Avanti Polar Lipids) and packed into a 3.2 mm rotor as reported in our previous protocols^17,22^. The temperature of the samples was held constant at 25 °C. The pulse sequence for recording 2D [^13^C,^13^C]-DARR experiment is shown in Fig. S7. All the critical parameters for the NMR experiments are given in Table S1.

### TIDE algorithm

For TIDE, a 2D FID time signal [S(t_1_, t_2_)], where t_1_ and t_2_ specifies indirect and direct time points, is treated as a complex matrix of size N_1_ × N_2_. The protocol consists of four different steps.

***Step 1***: The direct dimension of S(t_1_, t_2_) is Fourier transformed after appropriate window apodization. This step generates N_1_ number of 1D spectra S(t_1_, ω_2_). For the TIDE-processed spectra reported in the manuscript, we used an exponential apodization function with a line broadening of 50 Hz. In addition, zero-filling is applied in the direct dimension, with 2–4 k as the total time points.

***Step 2***: The FIDs in the t_1_ dimension are sliced into a series of sub-FIDs of length N, where N < N_1_ (Fig. S2). Typically, N is set to half of the total length of FID in the t_1_ dimension and can be optimized based on the spectral sensitivity and resonance life times. For the first set of sub-FID (S_1_), the t_1_ dimension ranges from 1 to N and for the i^th^ set of sub-FID (S_i_) from i to (N + i − 1),

$${{\rm{S}}}_{i}={\rm{S}}({{\rm{t}}}_{1}({\rm{i}}\,{\rm{to}}\,({\rm{N}}+{\rm{i}}-1)),{{\rm{\omega }}}_{2})$$

The first sub-FID, S_1_, contains the initial points of the parent FID, corresponding to the signals for all the resonances. As i increases, the relative intensity of long-lived resonances in the S_i_ increases; hence, it is possible to obtain a new dimension ‘i’ that encodes for the time-frequency information of the FID. The change in the relative intensities is represented in Fig. S13. As shown, two resonances (a, b) decay with transverse relaxation rates T_2_^a^ and T_2_^b^ respectively. When T_2_^a^ > T_2_^b^, the difference in the intensity of the magnetization initially increases, then decays. The rate of decay is faster for fast relaxing resonances.

***Step 3***: At this stage, the algorithm calculates the covariance for each sub-FID, S_i_:

$${{\rm{C}}}_{{\rm{i}}}({\omega }_{1},{\omega }_{2})=\text{Covariance}\,[{{\rm{S}}}_{i}]$$

Unlike Fourier transformation, the covariance operation does not introduce any first order phase artifacts in the spectrum. Note that the covariance operation here can be replaced with the calculation of Pearson correlation coefficients to generate a TIDE version of the cross correlation spectra^7^.

***Step 4***: At this point, *Step 3* of the protocol is repeated from i = 1 to n, generating a sequence of covariance spectra {*C*_*i*_} and the final TIDE-transformed spectrum is obtained by performing a Gaussian averaging of {*C*_*i*_} matrices:

$${p}^{th}\,plane\,of\,TIDE\,(TID{E}_{p})=\,\sum _{i=1}^{N}{C}_{i}({\omega }_{1},{\omega }_{2})\times {G}_{i}(p)$$

$${G}_{i}(p)=\frac{1}{{\rm{\sigma }}\sqrt{2{\rm{\pi }}}}{{\rm{e}}}^{-{({\rm{i}}-{\rm{p}})}^{2}/2{{\rm{\sigma }}}^{2}}$$

where *G*_*i*_(*p*), is the Gaussian function and σ^2^ is the variance. Since the first point of the *i* covariance spectra, {*C*_*i*_} is different, a Gaussian averaging of the {*C*_*i*_} is necessary to avoid covariance oscillations in the frequency response. The importance of this operation is illustrated in Fig. S3. Note that phase averaging reduces the offset dependent artifacts in the TIDE-processed spectra hence quantification is improved in the first plane of TIDE over original Covariance spectrum. Note that Gaussian averaging can be replaced by a Lorentzian function or other any other apodization functions. In general, the width of averaging function needs to be selected according to the life-time of the resonances and number of time points recorded in the indirect dimension. All the TIDE processing scripts are written in MATLAB^®^ (R2017a). The scripts are compatible with FID files generated by NMRPipe^41^. The total processing time for generating TIDE plane varies between 5 seconds to 1 min depending on the data size. In addition, the current scripts allow one to process selective spectral regions, reducing the total computational time. Average time for TIDE processing presented in this manuscript is 10 sec. using an Intel Core i7 processor and MATLAB^®^ R2017a. The output of TIDE processing is in the format of MATLAB figure, PDF, or jpg.

## Supplementary information

Supplementary file

Movie 1

## Data Availability

All the scripts and an example data set are provided on the following web site: http://veglia.chem.umn.edu/software-downloads/.
