# Supplementary material for: T2* weighted Deconvolution of NMR Spectra: Application to 2D Homonuclear MAS Solid-State NMR of Membrane Proteins
Source: Sci Rep. 2019 Jun 3;9:8225. doi: 10.1038/s41598-019-44461-3 (PMC6546711; doi:10.1038/s41598-019-44461-3)
Supplement: Supplementary file 1 — Supplementary file [file 41598_2019_44461_MOESM1_ESM.docx]

SUPPORTING INFORMATION

T_2_* weighted Deconvolution of NMR Spectra: Application to 2D Homonuclear MAS Solid-State NMR of Membrane Proteins

Manu V. S. ^1^, Tata Gopinath^1^, Songlin Wang^1^, and Gianluigi Veglia^1,2,*^

*^1^Department of Biochemistry, Molecular Biology and Biophysics, University of Minnesota, Minneapolis, MN, USA. ^2^Department of Chemistry, University of Minnesota, Minneapolis, MN, USA.*


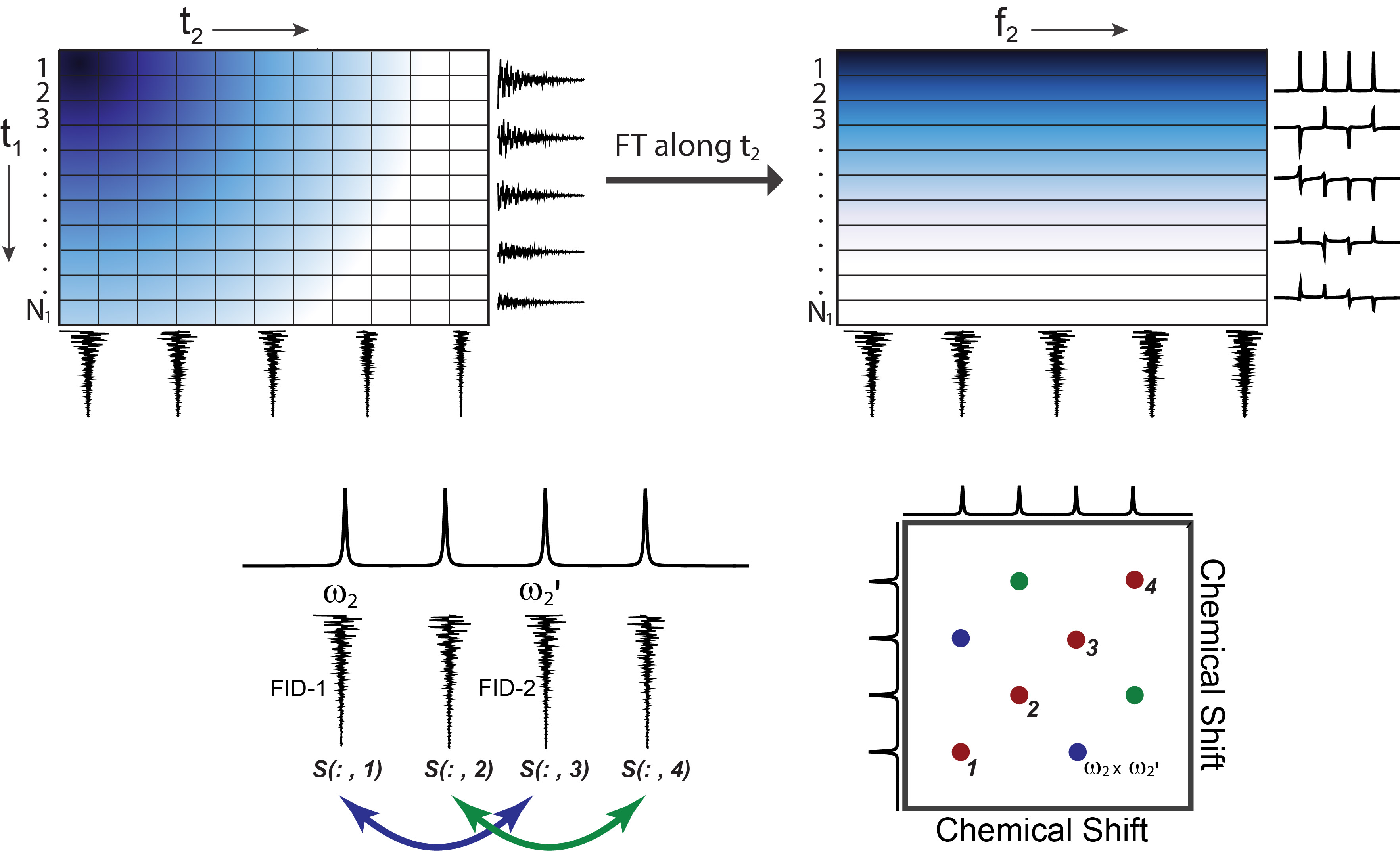


**Figure S1**. The schematic of 2D covariance NMR^1^ processing. The complex time matrix were Fourier transformed along $t_{2}$ followed by the Covariance evaluation step.


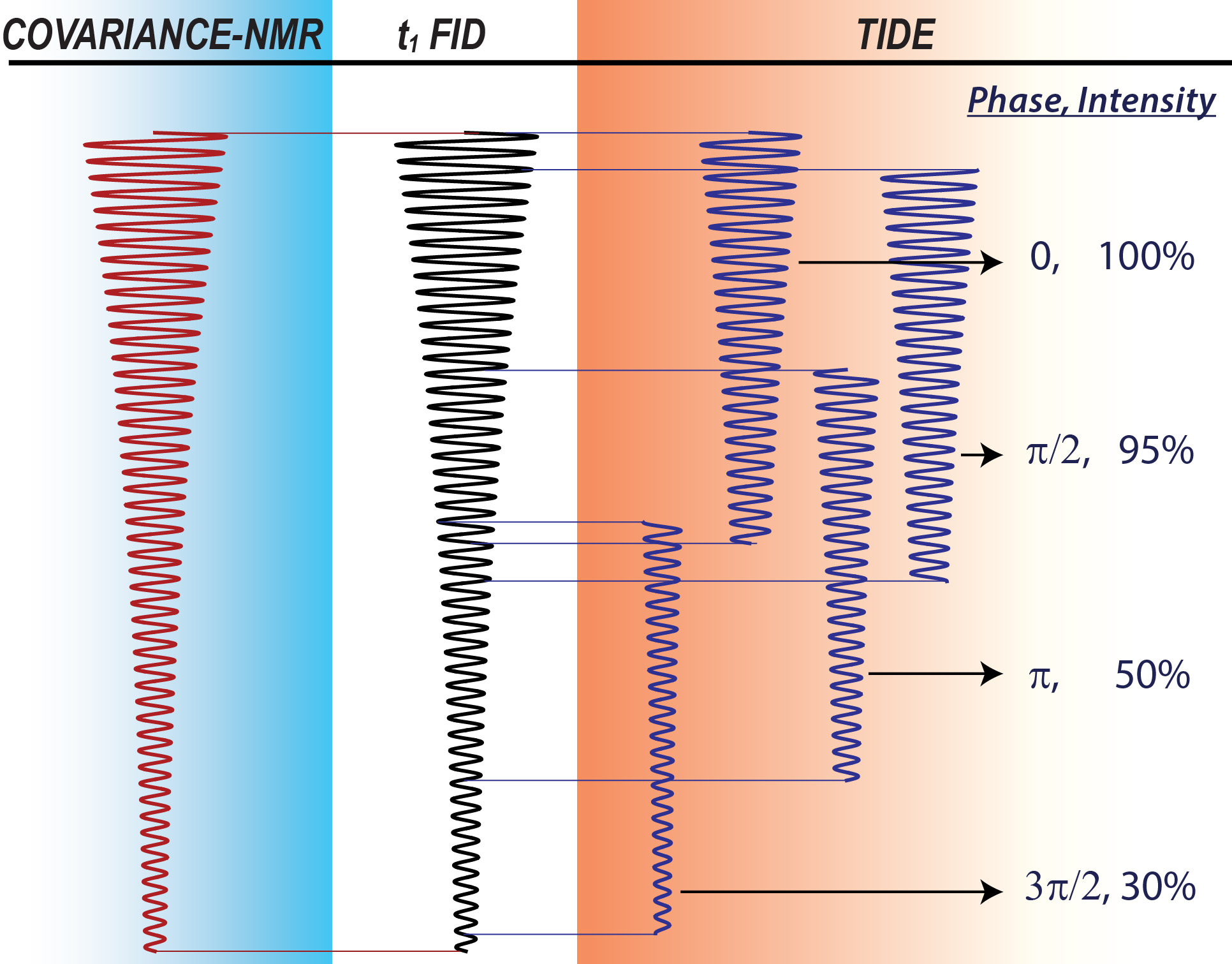


**Figure S2.** FID slicing step for TIDE processing. Conventional covariance NMR uses the full t_1_ FID points, whereas in TIDE processing, each FID is systematically sliced into sub-FIDs carrying the $T_{2}^{*}$ information.


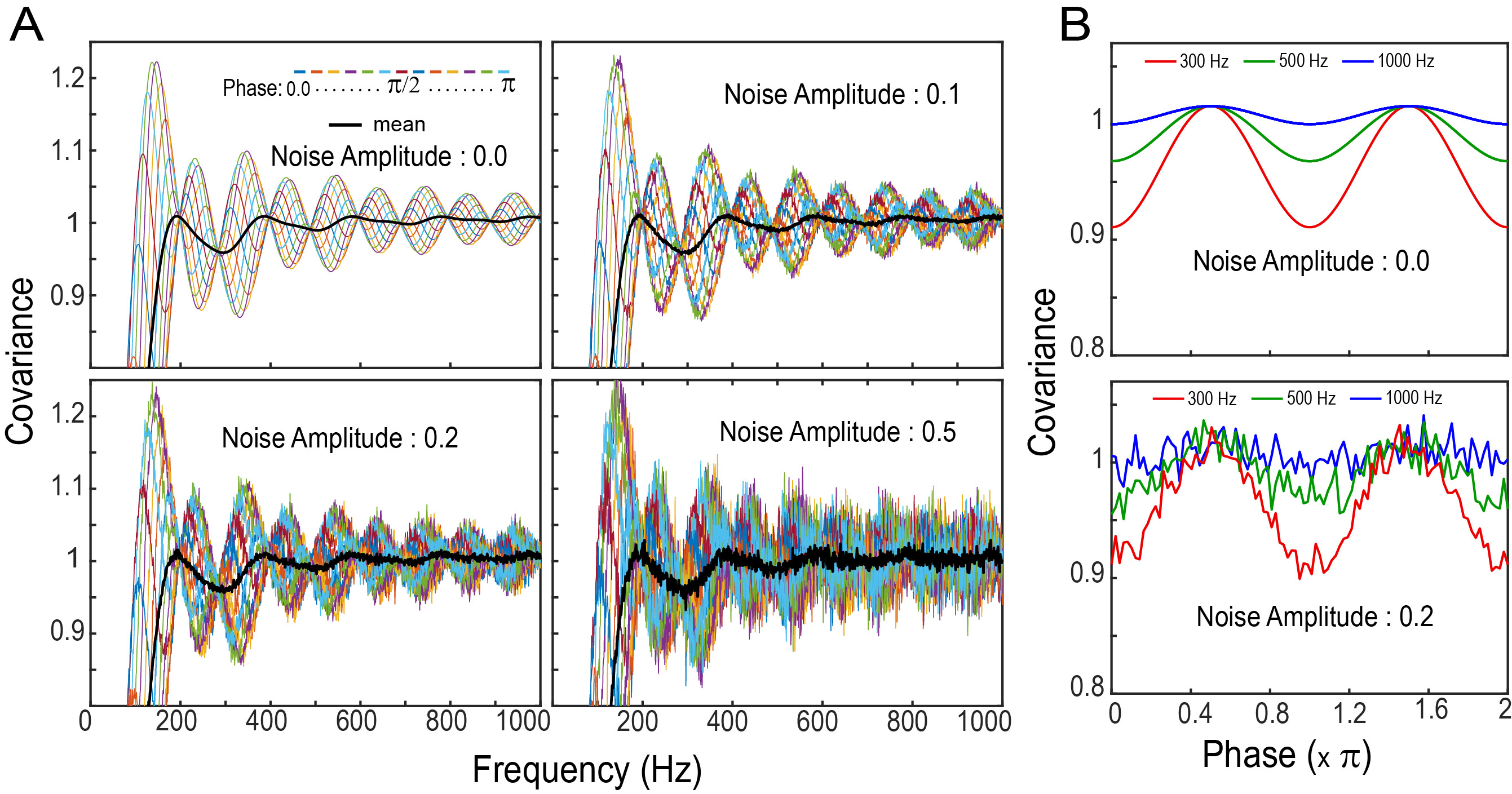


**Figure S3**: FID slicing generates sub-FIDs with different phases depending on the dwell time and the slice number. Here we simulated two 5ms (128 point) sine waves and evaluated the covariance at different phases. **A**. Covariance - frequency response of sine waves with 13 different phases sampled between 0 and π. The covariance-frequency oscillations are significantly reduced by averaging over different phases (black). The response curves are evaluated for noise amplitude levels 0, 0.1, 0.2 and 0.5, whereas signal amplitude is 1. **B**. The covariance oscillates with phase 300, 500, and 1000 Hz. With higher frequency, the FID packs more wave forms and hence the amplitude of the covariance oscillation is reduced and repeats when frequency close to the integer multiples of the Nyquist frequency.


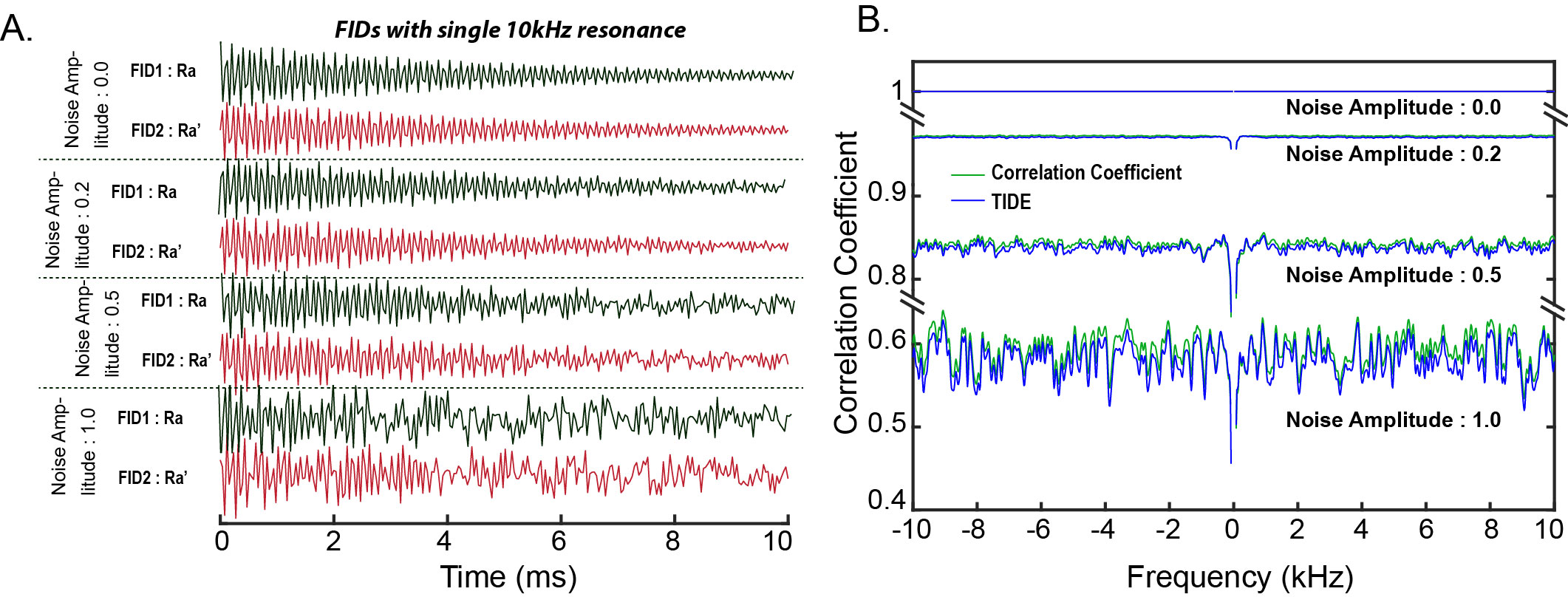


**Figure S4**. **A**. Simulated FIDs (FID-1, black, and FID-2, red) with single resonance (Ra = Ra’ = 10 kHz) and with 4 different noise amplitudes 0, 0.2, 0.5 and 1.0. Correlation coefficient is evaluated between FID-1 and FID-2 which differs only in noise profile. The frequency response is evaluated by varying the resonance frequencies of FID-1 (Ra) and FID-2 (Ra’) from -10 kHz to +10 kHz and are shown in **B**. The Correlation coefficient (green) and TIDE (blue) frequency responses do not introduce any frequency-dependent artifacts in the transformed spectra. The length of the FID used in the simulations was 10 ms and was sampled for 256 real points. For TIDE processing, the Gaussian averaging was performed over 10 covariance slices. Note that the two zero frequency FIDs with simulated noise levels does not correlate each other; therefore, in the middle of the response plot the covariance/TIDE is zero.


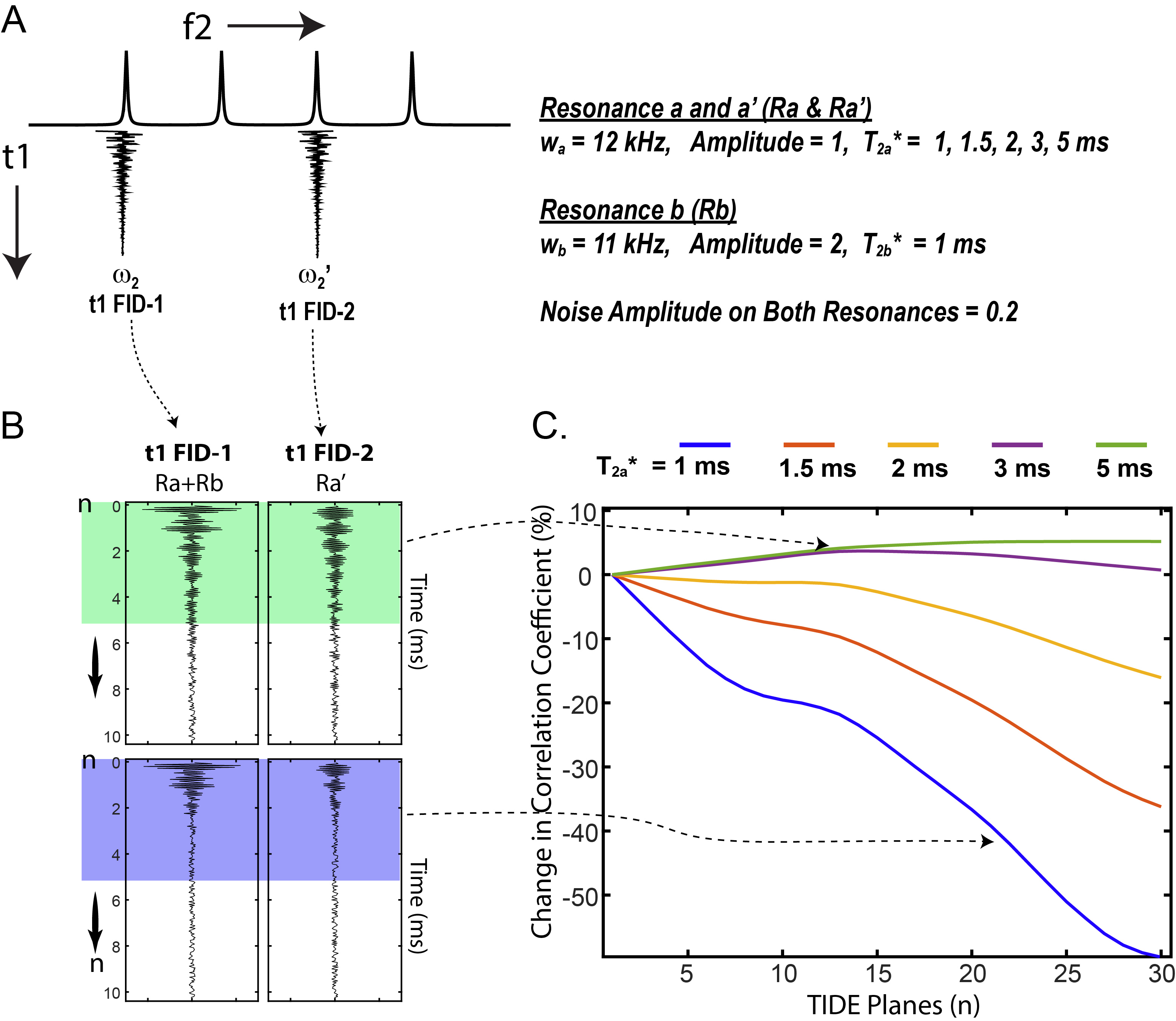


**Figure S5**. **A**. Two simulated *t*_1_ FIDs (FID-1 and FID-2) at positions ω_2_ and ω_2_’ of a 2D correlation data. Both FIDs contain *resonance-a* (12 kHz) with amplitude 1 and noise amplitude 0.2. FID -2 has an additional resonance (*resonance-b* at 11 kHz) with amplitude 2. **B**. FID-1 and FID-2 for long (5 ms, green) and short (1ms, blue) lifetimes of resonance-b. **C**. Change in correlation coefficient along the TIDE planes for T_2a_* relaxation rates 1, 1.5, 2, 3 and 5ms. With FID slicing moving down the *t*_1_ FID (shaded region in FIDs), the TIDE intensity response for short T_2_* (= 1ms) decrease, whereas in the case T2* = 5 ms, increases. The differences in peak heights increases as TIDE plane number increases as a result of the T_2_^*^.


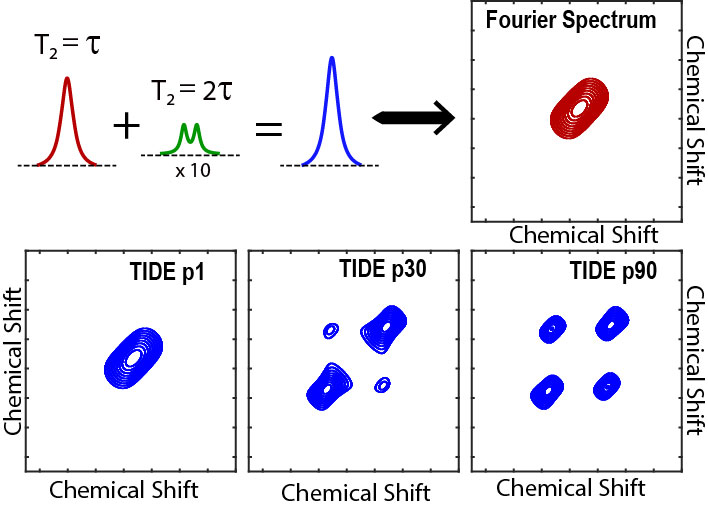


**Figure S6**. TIDE processing of a simulated homonuclear correlation data set including one intense short-lived peak with two long lived intense peaks which are correlated to each other. The Fourier spectra shows only the intense peaks where as in TIDE spectra, the long-lived peaks are resolved in higher planes.


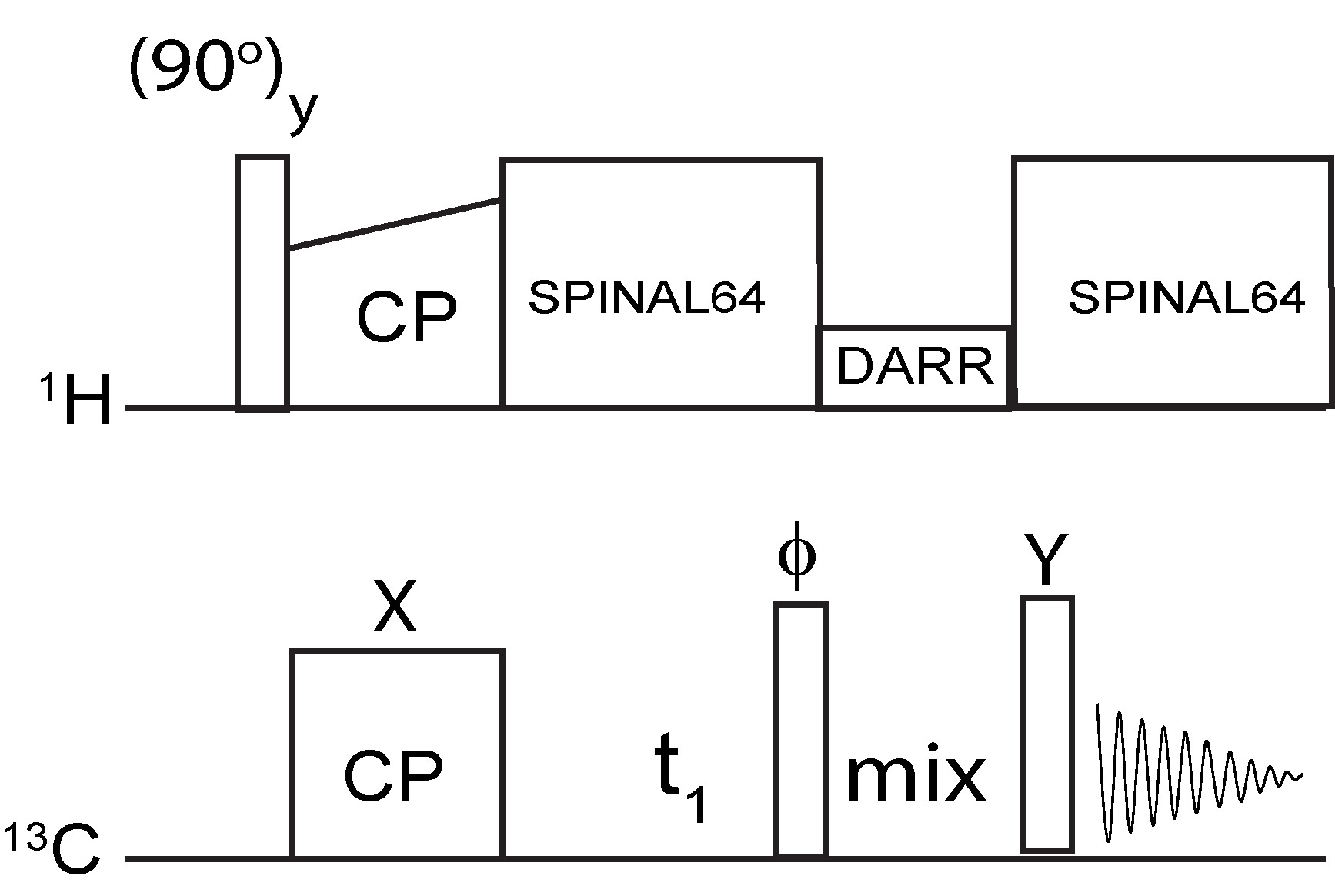


**Figure S7.** [^13^C,^13^C]-DARR pulse sequence. A two-step phase cycle was applied by switching the phase of initial θ^1^H pulse and receiver phase between y and -y. The nutation angle of θ is 90° for all DARR experiments except the NAVL sensitivity testing. The phase φ was altered between y and x for states mode t_1_ acquisition. Spinal64^2^ decoupling sequence is applied at ^1^H channel with power 100 kHz during direct (t2) and indirect (t_1_) acquisition time. DARR mixing time and CP time for all experiments mentioned in this manuscript are given in Table S1. DARR data acquired with this standard pulse sequence can process with TIDE for real and imaginary t_1_ points separately. Since the information content is same for real and imaginary t_1_ FIDs, TIDE averages the real and imaginary to improve S/N. A modification of this pulse sequence which acquire only the real points (as shown in Bruschweiler’s original paper^3^) can be used for reducing the experimental time by half.


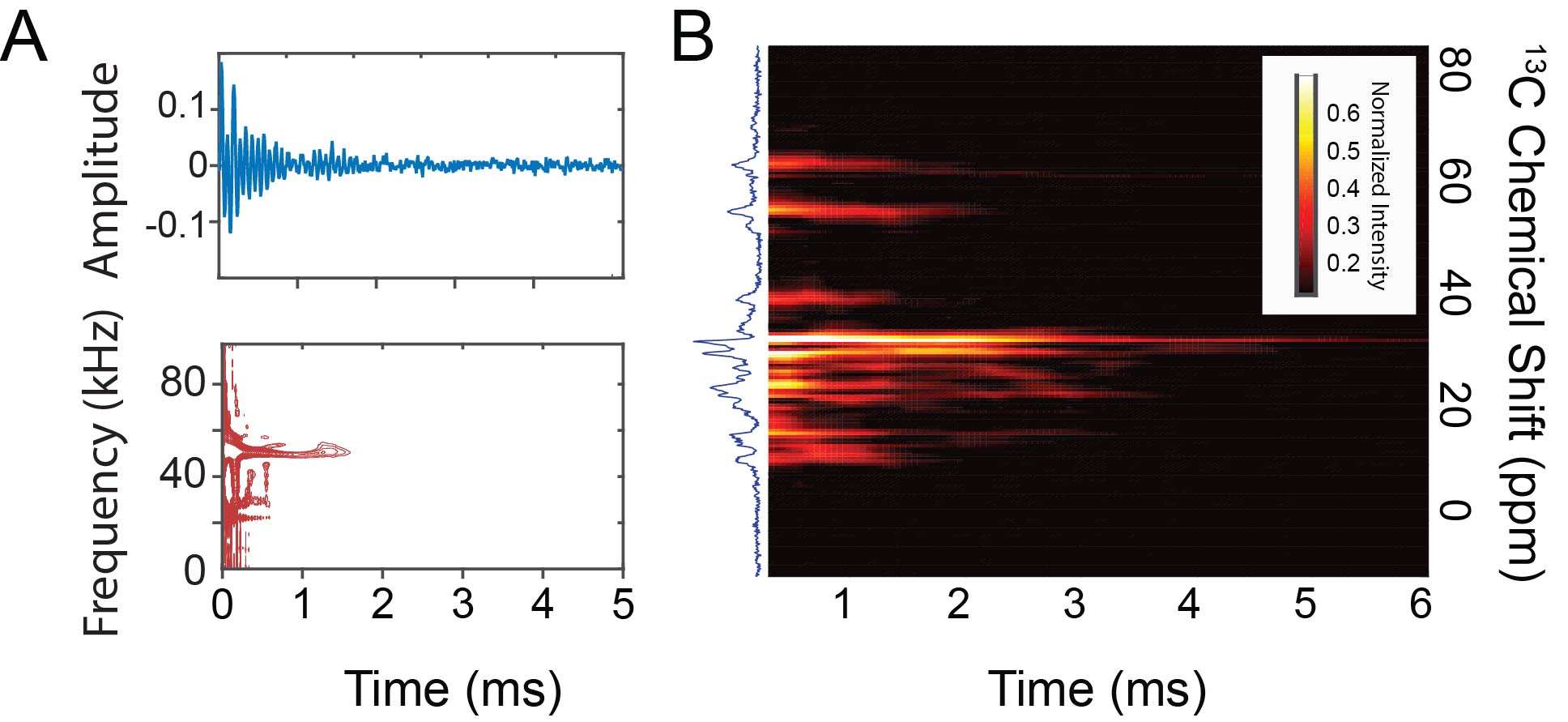


**Figure S8** Analysis of the frequency distribution for the [^13^C,^13^C]-DARR experiment of PLN in DMPC lipid bilayers. **A.** Reduced Interference Distribution (RID) spectrum (bottom) obtained using the toolbox from http://case.caltech.edu/tfr/. The FID used (top) corresponds to the first increment of the [^13^C,^13^C]-DARR experiment. **B**. Time-resolved 1D processing (pseudo-2D) of the first increment of [^13^C,^13^C]-DARR experiment.


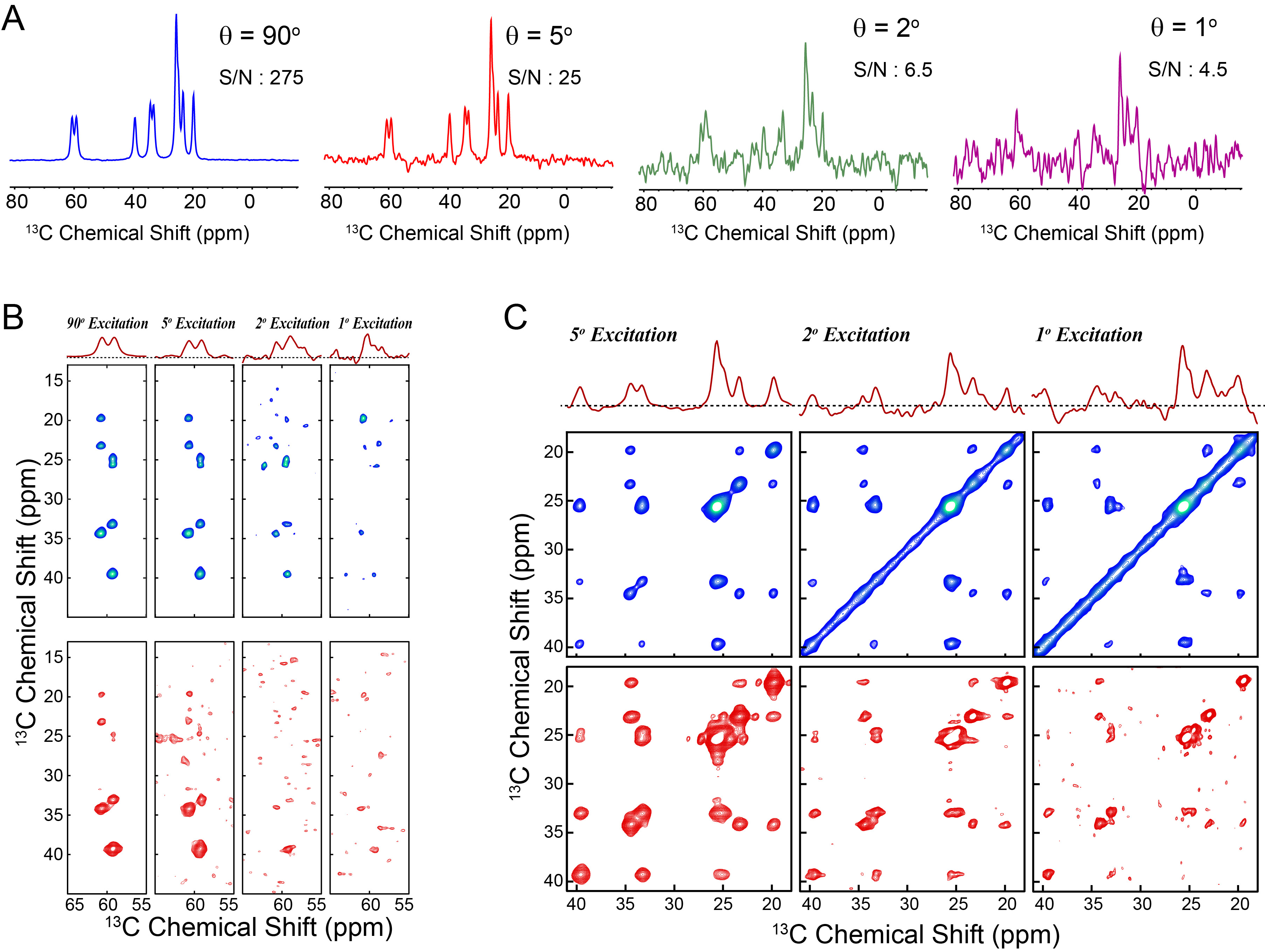


**Figure S9.** [^13^C,^13^C]-DARR experiments on NAVL (mixing time 10ms) by varying the angle (θ) of the initial excitation pulse on ^1^H channel. The relative sensitivity of spectra at θ = 5^o^, 2^o^ and 1^o^ are 8.5%, 3.5% and 1.8% of the maximum (θ = 90^o^) respectively. **A.** first 1D of DARR at different excitation angle. **B** and **C**. Fourier (red) and TIDE processed spectra (blue) of [^13^C,^13^C]-DARR at different sensitivity levels. TIDE processing makes it possible to detect the missing peaks in the FT [^13^C,^13^C]-DARR spectrum.


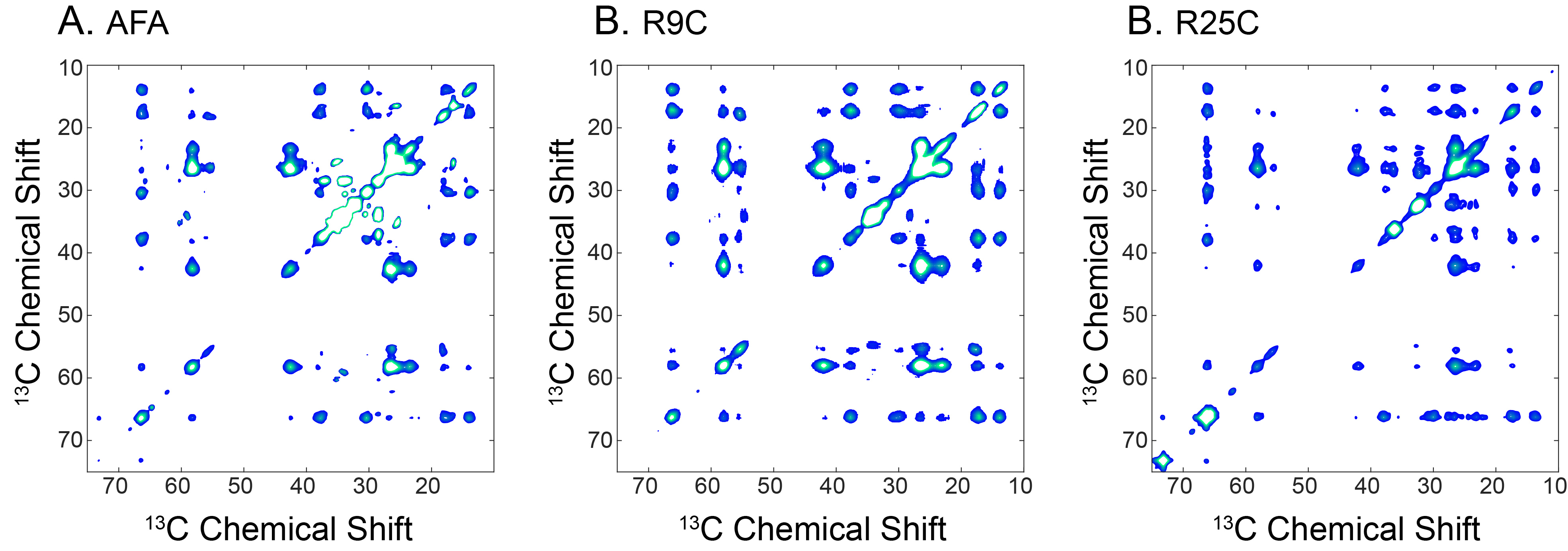


**Figure S10.** TIDE spectra of PLN and its mutants R9C and R25C. Experiments were performed on 700 MHz Varian spectrometer. The original data published previously and reported here with permission (copyright Elsevier) from Nelson *et al.*^4^.


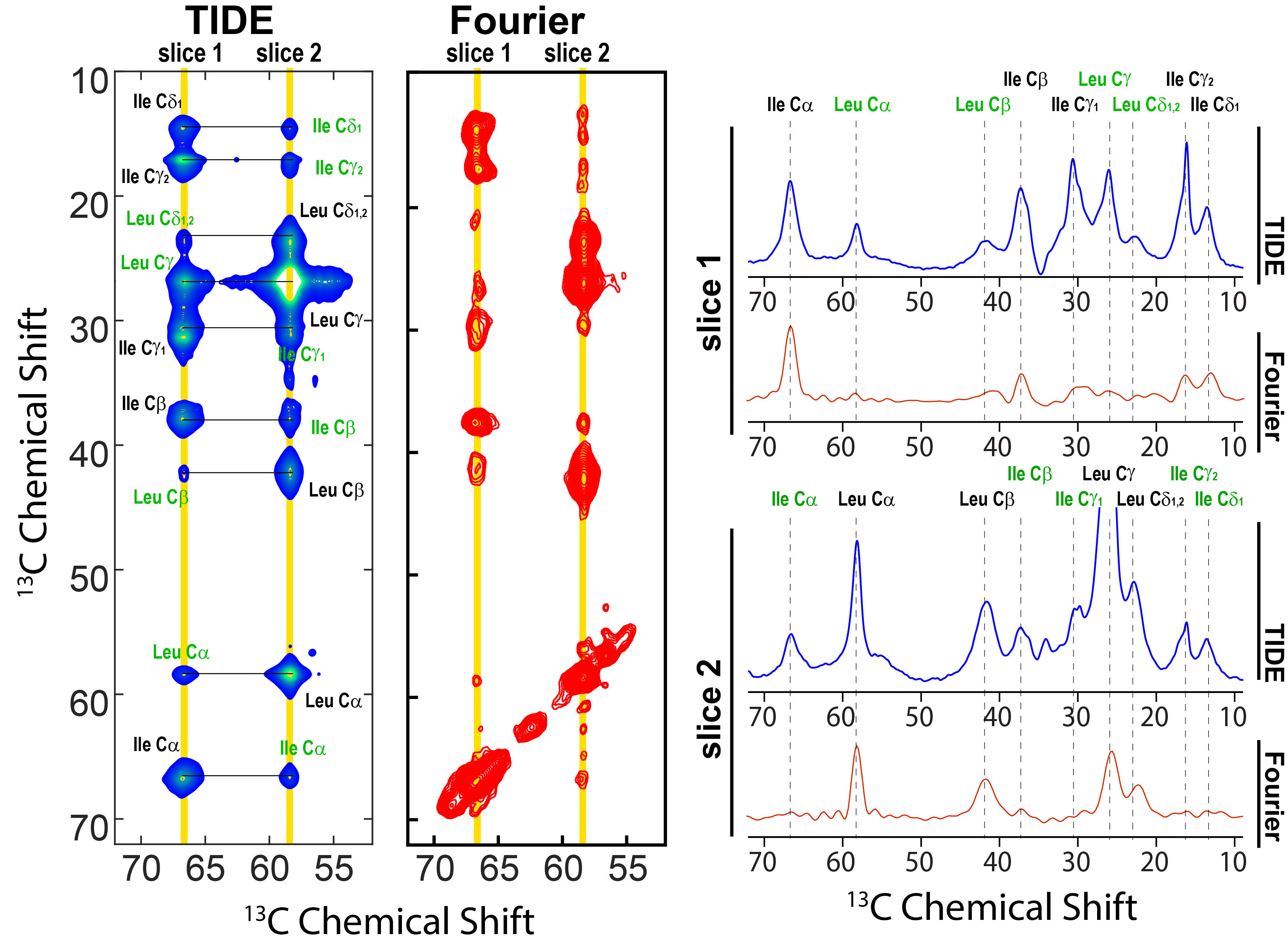


**Figure S11.** Fourier (red) and TIDE spectra (blue) of [^13^C,^13^C]-DARR experiments of U-^13^C-Leu / U-^13^C-Ile PLN pentamer at 200ms mixing time. Experiments were performed on 700 MHz Varian spectrometer. Intraresidue and interprotomer cross-peaks are labeled in black and green, respectively. The original data were adapted from Verardi *et al.^5^*.


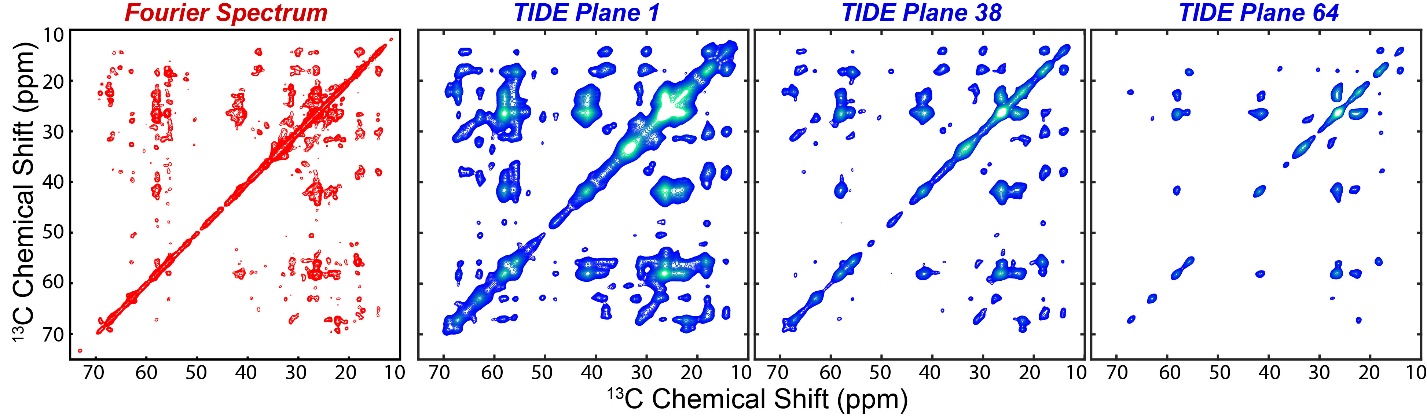


**Figure S12.** Fourier (red) and TIDE spectra (blue) of [^13^C,^13^C] DARR experiments of SATP protein at 100ms mixing time. Experiments were performed on 700 MHz Varian spectrometer at 298 K.


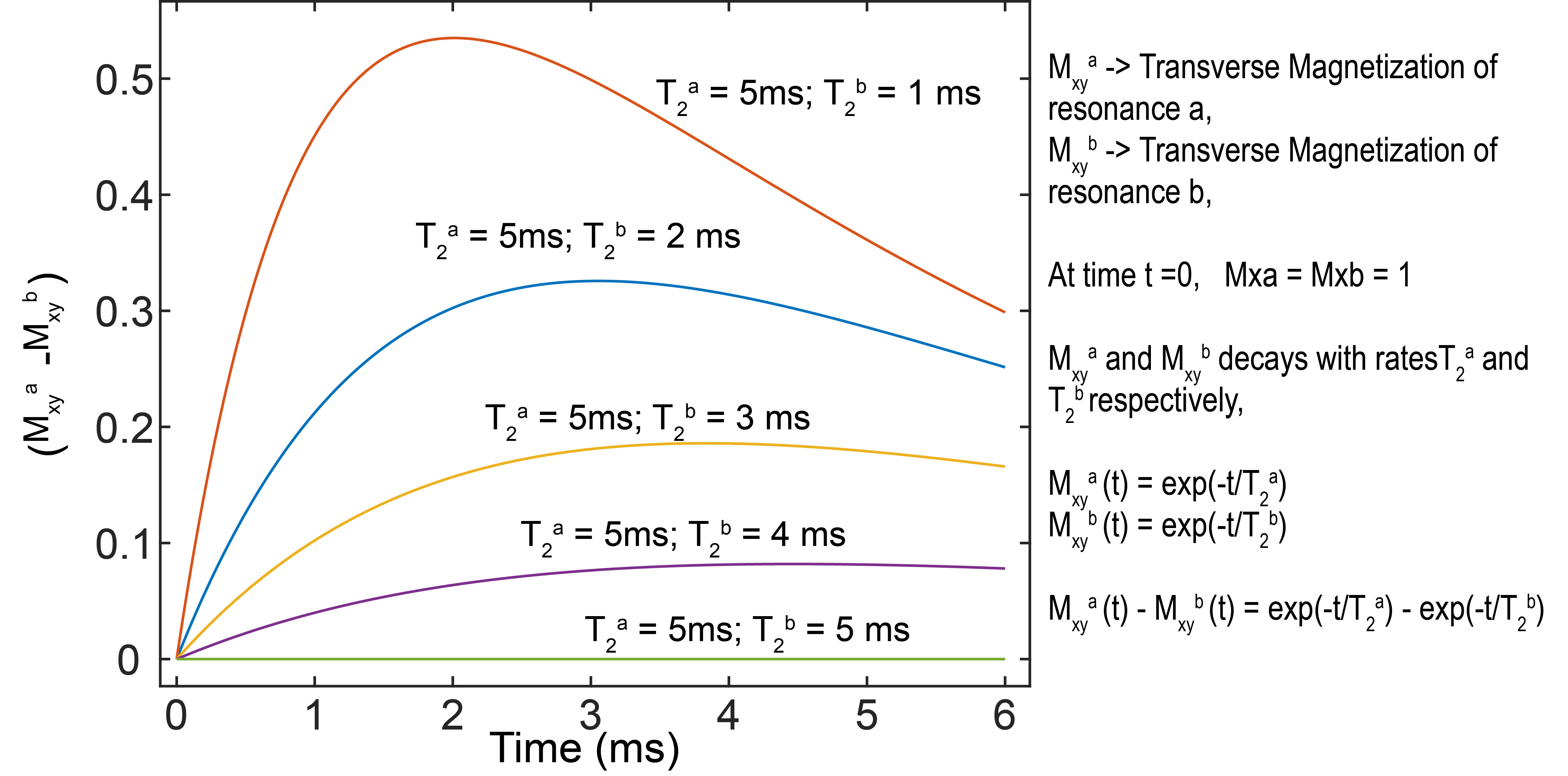


**Figure S13:** Relative changes in the intensities vs time for resonance a and b with different lifetimes (T_2_).

**Table S1**: Critical parameters for all the DARR experiments discussed in this manuscript.

| **Protein** | **Spectrometer** | **t1 points** | **Spinning (kHz)** | **CP mixing time**  **(μs)** | **DARR Mixing Time (ms)** | **Temperature (^o^C)** | **Figure** |
| --- | --- | --- | --- | --- | --- | --- | --- |
| **SLN** | Varian 600 MHz | 256 | 12 | 500 | 100 | 2 | 2 |
| **PLN AFA** | Bruker 600 MHz | 200 | 12 | 1000 | 100 | 25 | 3, S10 |
| **PLN** | Varian 700 MHz | 50 | 8 | 1000 | 200 | -25 | S11 |
| **PLN-R9C** | Varian 700 MHz | 150 | 12 | 1000 | 100 | 25 | 3, S10 |
| **PLN-R25C** | Varian 700 MHz | 150 | 12 | 1000 | 100 | 25 | 3, S10 |
| **SATP** | Varian 600 MHz | 256 | 12 | 500 | 100 | 2 | S12 |
| **NAVL** | Bruker 700 MHz | 1024 | 12 | 1000 | 10 | 25 | S9 |

**SUPPORTING REFERENCES**

1 Bruschweiler, R. Theory of covariance nuclear magnetic resonance spectroscopy. *Journal of Chemical Physics* **121**, 409-414, doi:10.1063/1.1755652 (2004).

2 Fung, B. M., Khitrin, A. K. & Ermolaev, K. An improved broadband decoupling sequence for liquid crystals and solids. *J Magn Reson* **142**, 97-101, doi:DOI 10.1006/jmre.1999.1896 (2000).

3 Bruschweiler, R. & Zhang, F. L. Covariance nuclear magnetic resonance spectroscopy. *Journal of Chemical Physics* **120**, 5253-5260, doi:10.1063/1.1647054 (2004).

4 Nelson, S. E. D. *et al.* Effects of the Arg9Cys and Arg25Cys mutations on phospholamban's conformational equilibrium in membrane bilayers. *Biochim Biophys Acta* **1860**, 1335-1341, doi:10.1016/j.bbamem.2018.02.030 (2018).

5 Verardi, R., Shi, L., Traaseth, N. J., Walsh, N. & Veglia, G. Structural topology of phospholamban pentamer in lipid bilayers by a hybrid solution and solid-state NMR method. *Proceedings of the National Academy of Sciences of the United States of America* **108**, 9101-9106, doi:10.1073/pnas.1016535108 (2011).
